# Determining the key elements of community-based care for women and partners following a stillbirth or second trimester miscarriage: protocol for a realist synthesis

**DOI:** 10.1136/bmjopen-2024-093581

**Published:** 2025-05-15

**Authors:** Becky MacGregor, Helen Leach, Rachel Court, Amy Grove, Alexander Edward Heazell, Sarah Hillman, Sophie Staniszewska

**Affiliations:** 1 Applied Health Sciences, University of Birmingham, Birmingham, UK; 2 Warwick Medical School, University of Warwick, Coventry, UK; 3 Birmingham Centre for Evidence and Implementation Science, University of Birmingham, Birmingham, UK; 4 Maternal and Fetal Health Research Centre, University of Manchester, Manchester, UK

**Keywords:** Postpartum Period, Pregnancy, Primary Health Care

## Abstract

**Abstract:**

**Introduction:**

Stillbirth and second trimester miscarriage have wide-reaching consequences for women, their partners and their families. There is currently little community-based care provision within the National Health Service for women and partners who have had a stillbirth or second trimester miscarriage. There is a research gap in the evidence to guide best practice. This review will seek to understand how, when and where this care is best delivered.

**Methods and analysis:**

The aim of this review is to identify the key elements of community-based care following a stillbirth or second trimester miscarriage. As this is a complex intervention, a realist approach will be used to establish for whom, how, when and where this care is best delivered. An initial programme theory will be constructed which will be tested against the available evidence and refined. A wide range of evidence including qualitative, quantitative, mixed-methods and experiential knowledge will be identified through secondary sources, extracted and synthesised to develop and refine the programme theory.

**Ethics and dissemination:**

This review will not require ethical approval as it does not involve primary data collection. The findings will be submitted for open-access publication to a peer-reviewed journal and disseminated to stakeholders.

**PROSPERO registration number:**

CRD42024587365.

**STRENGTHS AND LIMITATIONS OF THIS STUDY**

- The use of a realist review approach to explore this topic will allow inclusion of a wide range of evidence including case studies, charity reports, examples of good practice and experiential evidence.
- The inclusion of a co-production group in the review question development, theory development and synthesising the evidence will ensure applicability of the review findings.
- Theory development may be limited by the availability of relevant evidence.

## Introduction

 Stillbirth and second-trimester miscarriage are devastating life events for women and their partners. These events have important consequences for short-term and long-term, physical and mental health.^1 2^ Continuity and provision of care to address ongoing physical and psychological needs and provision of information to women and their partners about future pregnancy planning have been identified as key principles in caring for women who have experienced a pregnancy loss.^3 4^ However, there is currently little evidence regarding what community-based care following a stillbirth or second trimester miscarriage should include, who it should be provided for and in what setting it is best delivered. The purpose of this realist review is to fill this evidence gap.

### Stillbirth and second-trimester miscarriage in the UK

The stillbirth rate in the UK in 2021 was 3.54 per 1000 live births,^5^ which is an increase on previous years and equates to 2866 stillbirths.^6^ The annual rates of second trimester pregnancy loss are not routinely collected or reported in the UK, but it is estimated to be 1–2% of all ongoing pregnancies.^7^ Women from black, Asian and minority ethnic groups, and women from the most deprived areas of the UK are at increased risk of pregnancy loss.^5 8^ The rate of stillbirth in the UK among the socioeconomically most deprived women is 4.69 per 1000 live births, compared with 2.37 per 1000 live births for the least deprived.^5^ Black women in the UK are more than twice as likely to experience a stillbirth^5^ and have an increased risk of miscarriage when compared with white women.^8^ However, we do not know the anticipated effect of the intersectionality of these characteristics, which may lead to a compounding of inequality and discrimination.

### Consequences of stillbirth or second trimester miscarriage

Experiencing a stillbirth or second trimester miscarriage increases the risk of an adverse outcome in a later pregnancy. These risks include an increased risk of recurrent pregnancy loss, as well as preterm birth, pre-eclampsia or having a low-birthweight infant.^8^,^12^ There are associated short-term and long-term consequences for a woman’s health after a pregnancy loss, including increased risk of depression, cardiovascular disease and stroke.^813^,^15^ A recent report from the MBRRACE-UK (Mothers and Babies: Reducing Risk through Audits and Confidential Enquiries across the UK) Confidential Enquiry into Maternal Deaths highlighted the role that experiencing a pregnancy loss plays in maternal suicide.^15^ In addition to the direct effect on women’s physical and mental health, stillbirth and second trimester miscarriage have significant and substantial psychological, economic and societal consequences for the wider family and society.^1 8 16^

The experiences of partners following a stillbirth or second trimester miscarriage are less researched than those of birthing mothers.^17^,^21^ Targeted care for partners is currently not widely available in the UK,^17^ and provision of care for partners following a stillbirth or second trimester miscarriage is routinely overlooked.^19 22 23^ Partners may feel there is a lack of recognition of their loss from healthcare professionals, and this may compound their grief.^19^ Male partners are less likely to seek support following a pregnancy loss, but there is evidence that a good experience of the care received has a positive effect on the distress experienced.^19 24^ LGBTQ+partners may face additional barriers including prejudice and a lack of recognition of their parental rights.^23 25^

### Healthcare provision following stillbirth or second trimester miscarriage

In the UK, initial care following a stillbirth or second trimester miscarriage is delivered in a hospital setting, with any care delivered in community settings likely to be variable in content.^26^ This is similar to other high-income countries in Europe and Oceania.^27^ Current guidance from the Royal College of Obstetricians and Gynaecologists recommends an appointment in secondary care after a stillbirth or second trimester miscarriage.^28 29^ This appointment often has a clinical focus and ideally should occur in the first 3 months after a late pregnancy loss when the results of investigations are available. The appointment will review possible causes of the loss, arrange further tests if required and may discuss any modifiable risk factors for stillbirth or second trimester miscarriage.

*‘It feels like you fall off a cliff*’, following hospital discharge, is the phrase used by patient and public involvement and engagement contributors to describe community-based care following a stillbirth or second trimester miscarriage.^30^ Contributors talked about gaps in community-based care and the pervading feeling that women and partners feel their voices are lost and emotions and experiences are not validated.^30^ Women spoke repeatedly about a lack of community-based care provision and the difficulty they found proactively seeking care when grieving.^30^ Variability in community-based care provision, need for a care pathway and continuity of care to prevent women having to repeat their story multiple times were recurrent themes.^30^

### Community-based care following a stillbirth or second trimester miscarriage

Very little is known about what the role of General Practitioners (GPs) and other healthcare providers, including practice nurses, community midwives and health visitors, in delivering community-based care after a stillbirth or second trimester miscarriage in the UK and internationally.^27 31^ Community services including GPs are not currently a routine part of the patient journey following a stillbirth or second trimester miscarriage.^31^ The UK National Bereavement Care Pathway recognises that community-based staff including GPs and midwives are important sources of ongoing care after late pregnancy loss and that good follow-up care in the community is essential; there are no recommendations on what this care should consist of.^32^ What care should be offered, especially in relation to physical and long-term health, and when and how care is best delivered is also unclear.^33 34^

Community services including the National Health Service and third sector providers are ideally placed to deliver care after discharge into the community following a late pregnancy loss. GPs and the multidisciplinary teams they work with are a skilled workforce trained in providing holistic care to people with multimorbidity, including bereavement and mental health diagnoses. As these services are based in the communities in which women and their partners live, access barriers (eg, lack of transport) are reduced. Also, the need for women to return to hospital where the loss occurred, which may cause further distress, is negated. Community services may also have pre-existing relationships with women and partners which may help facilitate the provision of care.

The current gap in care and the lack of guidance on best practice are clear. There is a need to understand how, when and where community-based care is best delivered to women and partners following a stillbirth or second trimester miscarriage. We need to identify and understand the underlying causal mechanisms and contexts that may facilitate the delivery of community-based care which meets the needs of women and partners. In this realist review, we use the existing and available literature to establish for whom, how, when and where community-based care following a stillbirth or second trimester miscarriage is best delivered.

## Methods and analysis

**Figure 1:**
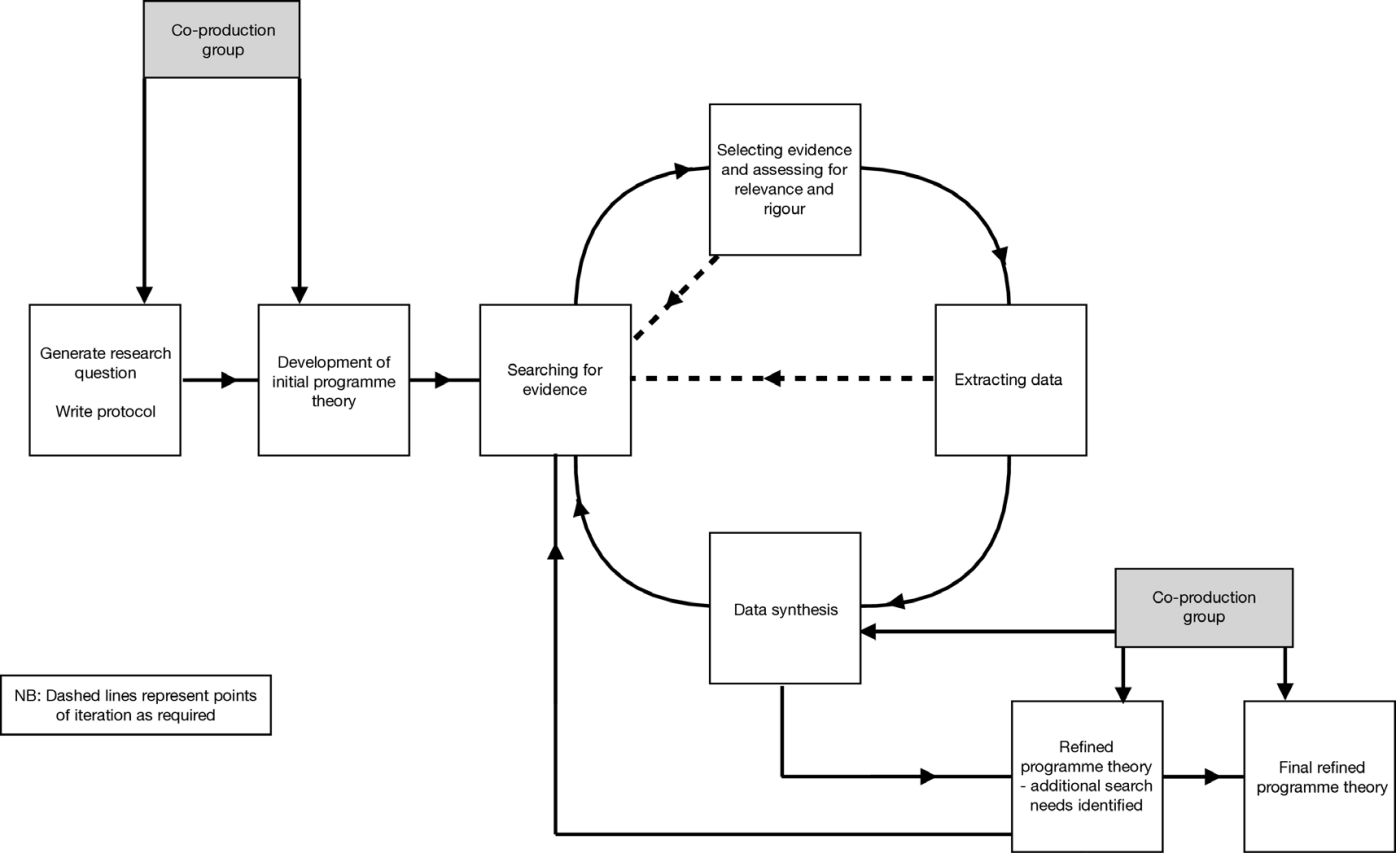
Flow diagram of the review process.

This review adopts realist review methodology which seeks to answer the question, ‘what works, why, for whom and in what circumstances?’ It will focus on understanding how interventions work in specific contexts.^35^ The review follows the principles set out by Pawson *et al*^36 37^ and will aim to establish what the key elements of community-based care are following a pregnancy loss and the mechanisms and contexts which enable their delivery. A realist review has been chosen because it allows for the inclusion of the grey literature and other broader types of evidence and is not limited to evidence generated from empirical studies. It also aims to provide an explanatory analysis of why care is best delivered in a particular way and how it might be implemented most effectively. A realist review is an iterative and interactive process,^36^ and this review will follow the recommended stages of literature searching, scoping, data inclusion, extraction and synthesis (see figure 1).^36^ The planned start date for the review is 1 April 2025, and the anticipated completion date is 1 April 2026. The final review synthesis will be written up using the RAMESES publication standards for realist synthesis.^37^

A co-production group of 10 relevant stakeholders, including women with lived experience (4), a partner with lived experience, healthcare professionals (2), a policy expert and representatives from third sector organisations (2), has been set up to partner with the researchers in carrying out the review. They are a diverse group of people who will bring their skills and knowledge to the review. This will include considering some of the issues and inequalities that affect underserved groups. Their expert opinion and experiential evidence will also be included as types of evidence.

Evidence may be drawn from comparable or similar areas of healthcare (see table 1), where concepts may have transferability, not just specifically from care provided following a stillbirth or second trimester miscarriage. (See online supplemental file 1 for key definitions) With this in mind, the review will initially explore five overlapping areas of research and practice to identify relevant evidence. These areas of scope are late pregnancy loss, community care, bereavement care, postnatal care and preconceptual care. These have been defined in table 1 and have been discussed and agreed by the co-production group. Evidence will be drawn from all areas and may intersect and overlap. Given the iterative nature of realist review methods, there is scope to explore and include other areas of research and practice as required.

**Table 1:** Areas that will be explored in the initial scope of the review

Late pregnancy loss	A stillbirth or second trimester miscarriage, this will include all types of current care following a late pregnancy loss, as well as women’s and partners' experiences of the care they receive,
Community-based care	Includes all care settings outside of a hospital, including care provided by third-sector organisations and charities, as well as community-based National Health Service settings, that provide care after a pregnancy.
Bereavement care	Supportive care that is provided to a family following the death of a child or any form of pregnancy loss including a termination of pregnancy or early miscarriage and will not be limited to bereavement care provided following a stillbirth or second trimester miscarriage.
Postnatal care	All care provided to women and their families postnatally. To include care that is provided after both a live birth and a pregnancy loss, as well as care provided in hospital and in the community and care provided by all healthcare professionals as well as by the voluntary sector.
Preconceptual care	Care that is provided prior to a pregnancy to identify and modify risk factors for adverse pregnancy outcomes and improve maternal health.

### Review objectives

1. To identify the key elements of community-based care following a stillbirth or second trimester miscarriage.
2. To establish for whom, how, when and where this care is best delivered.

### Review research question

For women and partners following a stillbirth or second trimester miscarriage, what contexts and mechanisms facilitate the key components of community-based care to achieve care that meets their needs?

### Formulating the question and constructing an initial programme theory

The review objectives and the review questions were generated based on gaps in current care and knowledge that were identified from discussion with stakeholders and patient and public involvement and engagement (PPIE) participants during a previous project which directly led to the current work^30^ and the expert knowledge of the review team. These were used to generate a review question by the lead reviewer and discussed with the review team. The question was then taken to the project co-production group for discussion and final agreement.

An initial programme theory will be generated using key concepts drawn from expert knowledge and from ideas generated by stakeholders and PPIE contributors.^30^ The initial programme theory will be taken to the project co-production group for further discussion, modification and final agreement.

A generative model of causality will be used to refine and test the initial programme theory. This means that the review will seek to identify relationships between the possible mechanisms (M) through which an outcome (O) occurs and the context (C) of this outcome.^36^ These models of causation are called CMO configurations, and through building, combining and revising these configurations, the programme theory will be refined.

### Conducting the background search and searching for programme theories

An initial scope of the literature using search terms to capture the initial areas of interest; ‘postnatal care’, ‘stillbirth’, ‘second trimester miscarriage’, 'bereavement care’, ‘preconceptual care’ and ‘community-based care’ with additional terms added and adjusted as needed will be undertaken. There will be no restriction on evidence type or study design; and documents including case studies, charity reports and examples of good practice will be included. The search strategy will incorporate a number of complementary search strategies that will be progressively extended and refined^38^ . These will include:

- Electronic database searching which may include EMBASE, MEDLINE, the Cochrane Library, PsycINFO, CINAHL, Web of Science and any other relevant databases that are identified.
- Literature scope using Google Scholar.
- Citation chaining and ‘related citations’ function on PubMed.^38^
- Snowballing; screening of reference lists from relevant papers to identify potential papers.
- Grey literature searches including national and international guidelines and published reports such as The Independent Pregnancy Loss Review^39^ and The Sands Listening Project.^40^

Searches will be limited to the English language and will initially focus on high-income countries with scope to extend beyond these countries as required if there are areas of practice identified that have transferability to a UK setting.

Two reviewers, with the help of an information specialist, will identify initial evidence that is deemed relevant and provides context. Pawson *et al* have described how this stage often involves ‘*locating the administrative thinking, policy history, legislative background, key points of contention in respect of the intervention’*.^36^ Identified evidence will be used to build and modify CMO configurations that will be combined and refined to generate theories which support or refute the initial programme theory.^36^ The CMO configurations and programme theories generated will be taken to the co-production group for discussion before searching for further empirical evidence to support or refute the modified programme theory.

### Searching for empirical evidence and refining the programme theory

Further searches will then be carried out to further identify empirical evidence that can be used to support, refute and refine the programme theory.^38^ This more focused search of the evidence and databases may include using search terms linked to the CMO configurations and programme theories as well as a combination of the other search methods already listed. Again, empirical evidence will be taken to the co-production group for discussion, to draw on their experiential knowledge, to test the evidence and to refine the programme theory further.

The evidence identification and selection process will be iterative and may require multiple cycles of searching for evidence, then testing and refining the CMO configurations and programme theories to create the final programme theory. The final programme theory will be agreed with the co-production group.

### Recording the search process

Evidence will be collated using the review management software Covidence or similar package and this will generate an electronic record of evidence searched and those excluded and included. A record of databases searched and any other sources used will also be created in order that this can be reported as part of the final review.

### Data extraction

Data extraction will take the form of assessing data for evidence to confirm or refute the programme theory and then making notes, highlighting or annotating the relevant sections of the data.^36^ A data extraction form will be created that is unique to this review and will be mapped to the CMO configurations and programme theories to be tested and refined.^41^ The extracted data will be collated using Logseq or a similar programme. A record will be kept by Logseq of the data sources used and the order in which data were extracted. The data may be returned to more than once, and as it is extracted, it will be assessed for relevance and rigour.^42^

### Data synthesis

The aim of data synthesis is to interrogate theory and refine it to generate a final programme theory. This is done through identifying evidence and comparing it with the programme theory to confirm or refute it and then refine it. The synthesis in this review aims to understand and explain what the key components of community-based care are for women and partners who have experienced a stillbirth or second trimester miscarriage, in what setting, to whom and how they should be delivered. These key components may include items such as the timing, setting and content of community-based care.

Evidence will be assessed for its methodological strengths by considering the relevance and rigour of the data.^36^ In the context of this review, data relevance considers whether the study or piece of evidence covers the theory that is being tested.^36^ Rigour refers to how trustworthy and plausible the data are.^36^

Possible stages in the synthesis process include^43^:

- Juxtaposing sources of evidence: this involves pairing sources of evidence that may have the same outcome but different contexts and mechanisms. For example, one source of evidence may have information about timing while another may have information about setting, with both having outcomes of better care.^43^
- Reconciling sources of evidence: this involves looking for evidence for circumstances that may hinder the delivery of an intervention, not just for circumstances that support it. Evidence that may help with this includes situations where outcomes differ in similar settings; these will be examined for possible explanations.^43^
- Consolidating and situating evidence: this involves comparing situations where the same outcome is achieved to look for similarities and determine if the evidence is sufficient to identify patterns across the evidence or whether there are differences in the mechanisms through which the outcomes are achieved.^43^ This will help to give a better understanding of the facilitators and inhibitors to delivering care.

### Patient and public involvement

The objectives and research questions for this review were discussed and agreed with the project co-production group. They will be involved in developing the programme theory, weighing the evidence and agreeing the final programme theory as detailed in the protocol. The co-production group will also be involved in creating dissemination materials for a lay audience and disseminating findings to the project stakeholders.

## Ethics and dissemination

This review does not require ethical approval as no primary data will be collected or used. Research ethics approval will be obtained before the next stage of the project.

The refined programme theory from this review will be taken forward to be further tested and refined by gathering primary data through indepth interviews with women and partners with lived experience and discussion with healthcare professionals. Sampling for interviews will be purposive and with particular emphasis on including women and partners from Asian, black and minoritised ethnic groups and those who are socioeconomically disadvantaged. At the end of primary data collection, a final programme theory will be generated. The final programme theory will be used to co-produce an intervention to meet the needs of families who have experienced a stillbirth or second trimester miscarriage.^44^ The finished review will be submitted for publication as an open-access article in a peer-reviewed journal with a focus on community-based care or women’s health and the findings presented at conferences. Results will also be disseminated to the project stakeholders in a format designed for a lay audience which may take the form of a plain English report or infographic.

## Supplementary material

10.1136/bmjopen-2024-093581 online supplemental file 1
