# Supplementary material for: Determining the key elements of community-based care for women and partners following a stillbirth or second trimester miscarriage: protocol for a realist synthesis
Source: BMJ Open. 2025 May 15;15(5):e093581. doi: 10.1136/bmjopen-2024-093581 (PMC12083345; doi:10.1136/bmjopen-2024-093581)
Supplement: online supplemental file 1 [file bmjopen-15-5-s001.docx]

**Appendix**

*Terms of reference*

This protocol refers to women, however the concepts herein apply to all birthing people.

**Stillbirth:** a baby delivered with no signs of life known to have died after 24 completed weeks of pregnancy [29].

**Second-trimester miscarriage:** a spontaneous pregnancy loss that occurs between 13- and 23+6-weeks’ gestation

**Postnatal period**: the time from the day of delivery to one year after birth
