# Supplementary material for: Determining the key elements of community-based care for women and partners following a stillbirth or second trimester miscarriage: protocol for a realist synthesis
Source: BMJ Open. 2025 May 15;15(5):e093581. doi: 10.1136/bmjopen-2024-093581 (PMC12083345; doi:10.1136/bmjopen-2024-093581)
Supplement: Supplementary file 2 [file bmjopen-2024-093581.reviewer_comments.pdf]

PEER REVIEW HISTORY

BMJ Open publishes all reviews undertaken for accepted manuscripts. Reviewers are asked to complete a checklist review form and are provided with free text boxes to elaborate on their assessment. These free text comments are reproduced below.

ARTICLE DETAILS

Title (Provisional)

Determining the key elements of community-based care for women and partners following a stillbirth or second trimester miscarriage: protocol for a realist synthesis

Authors

MacGregor, Becky; Leach, Helen; Court, Rachel; Grove, Amy; Heazell, Alexander Edward; hillman, sarah; Staniszewska, Sophie

---

VERSION 1 - REVIEW

**Reviewer**	**1**
**Name**	**Henry, Carrie J.**
**Affiliation**	**The University of Alabama System**
**Date**	**05-Dec-2024**
**COI**	**None**

Thank you for the opportunity to review this interesting protocol. The authors have identified an important gap in our knowledge of best practices in community-based care of women and families following pregnancy loss. The methods described are appropriate for filling the identified gap and providing sorely-needed guidance to practitioners, policy-makers, and researchers alike. I very much look forward to reading the final review.

I noticed a few typos:

P.4, line 16 I believe the sentence should begin with "Although."

P. 6, line 50 "using the" appears twice in the sentence.

P. 7, line16 "its" does not need an apostrophe.

P. 11 This figure is helpful in understanding the review methods, but I did not see it referred to in the text.

**Reviewer**	**2**
**Name**	**Rayment-Jones, Hannah**
**Affiliation**	**King's College London, Women and Children's Health**

**Date**	**23-Feb-2025**
**COI**	**None**

Thank you for the opportunity to review this well-structured and clearly articulated protocol, which presents a strong rationale and an appropriate methodology. The background section highlights inequalities affecting certain groups, so it would be helpful to clarify how these will be addressed within the methodology. For example, is the co-production group sufficiently diverse to comprehensively inform the development of the programme theory? Another consideration is ensuring clarity around the scope—will the search focus solely on UK-based NHS care, or will it extend to international examples of community-based care for women and partners following stillbirth or second-trimester miscarriage? Additionally, discussing the availability of relevant evidence, including grey literature, would help justify the choice of a realist review approach

VERSION 1 - AUTHOR RESPONSE

Reviewer: 1

Comments to the Author:
Thank you for the opportunity to review this interesting protocol. The authors have identified an important gap in our knowledge of best practices in community-based care of women and families following pregnancy loss. The methods described are appropriate for filling the identified gap and providing sorely-needed guidance to practitioners, policy-makers, and researchers alike. I very much look forward to reading the final review.

Thank you for this positive feedback.

I noticed a few typos:
P.4, line 16 I believe the sentence should begin with "Although."

We were unable to identify which sentence this related to.

P. 6, line 50 "using the" appears twice in the sentence

We have corrected this typo.

P. 7, line16 "its" does not need an apostrophe.

We have removed this apostrophe.

P. 11 This figure is helpful in understanding the review methods, but I did not see it referred to in the text.

We have included a reference to the figure in the text.

Reviewer: 2

Comments to the Author:

Thank you for the opportunity to review this well-structured and clearly articulated protocol, which presents a strong rationale and an appropriate methodology.

Thank you for this positive feedback.

The background section highlights inequalities affecting certain groups, so it would be helpful to clarify how these will be addressed within the methodology. For example, is the co-production group sufficiently diverse to comprehensively inform the development of the programme theory?

Thank you for your question. Our co-production group includes a diverse group of people, and this will enable us to interpret the evidence and draw conclusions by incorporating a variety of viewpoints to address some of the issues that affect underserved groups. We anticipate that there may be limited secondary evidence sources available to address some of these questions and we plan to build any evidence gaps identified into the next stage of the evaluation in which we will gather primary evidence to further build on and refine the programme theory generated by the review. Additional information addressing this has been added in to the protocol in the methods and dissemination section.

Another consideration is ensuring clarity around the scope—will the search focus solely on UK-based NHS care, or will it extend to international examples of community-based care for women and partners following stillbirth or second-trimester miscarriage?

Many thanks for your comment. The focus of the search will initially be on high-income countries to maximise transferability to a UK setting with scope to extend beyond these countries as required if there are areas of practice identified that have transferability to a UK setting. This information has been included in the study protocol.

Additionally, discussing the availability of relevant evidence, including grey literature, would help justify the choice of a realist review approach

Thank you for your comment. A realist review has been chosen because it allows for the inclusion of grey literature and wider evidence and is not limited to evidence generated from empirical studies which is important to given that there is a current gap in the available empirical evidence as to how best to deliver care to this group of women. This rationale has been added to the protocol and the availability of relevant evidence has been highlighted.
